# Hospital-acquired influenza infections detected by a surveillance system over six seasons, from 2010/2011 to 2015/2016

**DOI:** 10.1186/s12879-020-4792-7

**Published:** 2020-01-28

**Authors:** P. Godoy, N. Torner, N. Soldevila, C. Rius, M. Jane, A. Martínez, JA. Caylà, A. Domínguez, the Working Group on the Surveillance of Severe Influenza Hospitalized Cases in Catalonia, M. Alsedà, J. Álvarez, C. Arias, P. J. Balañà, I. Barrabeig, N. Camps, M. Carol, J. Ferràs, G. Ferrús, N. Follia, P. Godoy, P. Bach, M. Jané, A. Martínez, S. Minguell, I. Parrón, E. Plasència, M. R. Sala-Farré, N. Torner, R. Torra, J. Torres, JA. Caylà, P. Gorrindo, C. Rius, M. A. Marcos, M. D. M. Mosquera, A. Vilella, A. Antón, T. Pumarola, M. Campins, D. García, E. Espejo, N. Freixas, M. Riera Garcia, E. Maraver, D. Mas, R. Perez, J. Rebull, J. Pou, G. García-Pardo, M. Olona, F. Barcenilla, D. Castellana, G. Navarro-Rubio, L. L. Force

**Affiliations:** 1 Epidemiology Service. Agència de Salut Pública de Catalunya, Generalitat de Catalunya, Barcelona, Spain; 2 CIBER de Epidemiología y Salud Pública (CIBERESP), Barcelona, Spain; 3 Institut de Recerca Biomédica de Lleida, IRBLleida, Lleida, Spain; 4 Departamento de Medicina, Universidad de Barcelona, Barcelona, Spain; 5 Agència de Salut Pública de Barcelona, Barcelona, Spain

**Keywords:** Healthcare-associated infection, Hospitalized patients, Influenza, Nosocomial infection

## Abstract

**Background:**

In addition to outbreaks of nosocomial influenza, sporadic nosocomial influenza infections also occur but are generally not reported in the literature.

This study aimed to determine the epidemiologic characteristics of cases of nosocomial influenza compared with the remaining severe cases of severe influenza in acute hospitals in Catalonia (Spain) which were identified by surveillance.

**Methods:**

An observational case-case epidemiological study was carried out in patients aged ≥18 years from Catalan 12 hospitals between 2010 and 2016. For each laboratory-confirmed influenza case (nosocomial or not) we collected demographic, virological and clinical characteristics. We defined patients with nosocomial influenza as those admitted to a hospital for a reason other than acute respiratory infection in whom ILI symptoms developed ≥48 h after admission and influenza virus infection was confirmed using RT-PCR. Mixed-effects regression was used to estimate the crude and adjusted OR.

**Results:**

One thousand seven hundred twenty-two hospitalized patients with severe laboratory-confirmed influenza virus infection were included: 96 (5.6%) were classified as nosocomial influenza and more frequently had > 14 days of hospital stay (42.7% vs. 27.7%, *P* < .001) and higher mortality (18.8% vs. 12.6%, *P* < .02). The variables associated with nosocomial influenza cases in acute-care hospital settings were chronic renal disease (aOR 2.44 95% CI 1.44–4.15) and immunodeficiency (aOR 1.79 95% CI 1.04–3.06).

**Conclusions:**

Nosocomial infections are a recurring problem associated with high rates of chronic diseases and death. These findings underline the need for adherence to infection control guidelines.

## Background

Each year, 5–20% of the population are infected by the influenza virus [1]. Among adults aged ≥65 years and patients with underlying chronic diseases influenza is a leading cause of severe illness and death. Sentinel surveillance of patients hospitalized due to severe laboratory-confirmed influenza is critical to control the timing and spread of influenza and detect variations in circulating influenza viruses [2, 3].

The Department of Health of Catalonia (Spain) introduced the surveillance of patients hospitalized with laboratory-confirmed influenza to supplement the information provided by the influenza sentinel surveillance system, based on primary healthcare physicians, in October 2010 [4]. During periods of increased influenza activity in the community and the subsequent increase in the number of patients with influenza in hospitals, the risk of nosocomial transmission from infected patients increases [5].

Nosocomial influenza is a recognized public health problem in acute-care hospital settings [5] and has been associated with significant morbidity, mortality and high economic costs due to longer hospital stays. It is likely to be under-recognized due to rapid patient turnover and delays in diagnosis.

Outbreaks of influenza have been reported in different hospital wards [6–11]. In addition, sporadic nosocomial influenza infections may occur but are generally not identified or reported [5].

This study aimed to determine the epidemiologic characteristics of nosocomial influenza cases in acute-care hospital settings in Catalonia (Spain) identified by surveillance rather than through outbreak control activity during six consecutive influenza seasons (2010–2011 to 2015–2016).

## Methods

### Epidemiological study

The general methods of this study have been published elsewhere [12, 13]. In summary, we conducted an observational case-case epidemiological study of the characteristics and risk factors of nosocomial influenza cases in patients hospitalized due to laboratory-confirmed influenza in acute-care hospital settings.

Catalonia (Spain) initiated, in 2010, the surveillance of patients hospitalized due to laboratory-confirmed influenza. The system included a catchment area of 4,644,543 persons. During each influenza season, the 12 hospitals included in the surveillance system report on patients hospitalized with severe laboratory-confirmed influenza admitted to one of these hospitals.

### Study population and data collected

The study population was reported cases aged ≥18 years hospitalized with severe laboratory-confirmed influenza virus infection during six influenza seasons (2010–2011 to 2015–2016). We included patients hospitalized ≥24 h in any participating hospitals who presented an influenza-like-illness (ILI). A sentinel physician who screened all patients with severe ILI enlisted patients in each participating hospital. All patients had a nasopharyngeal or throat swab (bronchoalveolar lavage fluid or tracheal aspirate for intensive care unit (ICU) patients) and influenza virus infection was detected using reverse transcription polymerase chain reaction (RT-PCR).

From this source of acute-care hospital influenza patients, we defined patients with nosocomial influenza as those admitted to a hospital for a reason other than acute respiratory infection in whom ILI symptoms developed ≥48 h after admission and influenza virus infection was confirmed using RT-PCR.

A structured questionnaire was used by public health officers to collect data from each reported case by interview and review of medical records. This included socio-demographic data, obesity (body mass index [BMI] > 40), pregnancy, major chronic conditions (chronic obstructive pulmonary disease [COPD]), diabetes, chronic renal disease, immunodeficiency (HIV infection or other), chronic cardiovascular disease, and chronic liver disease).

### Laboratory data

Patient samples were first tested in the laboratories of the participating hospitals using an in-house real-time RT-PCR for influenza A and B after manual nucleic acid extraction. Samples with unsubtyped influenza virus were sent to the Catalan Influenza Reference Laboratory to determine the subtype. Molecular subtyping was used to determine the H subtype for influenza A and the lineage for influenza B. Subtyping failed in some cases due to a low viral load and such samples were classified as “unidentifiable”.

### Statistical analysis

We compared the baseline characteristics of nosocomial influenza cases with those of other hospitalized cases of severe influenza. The baseline variables considered were: sex, age (18–64, 65–74 and ≥ 75 years), virus type, major chronic conditions (COPD, diabetes, obesity [BMI > 40], chronic renal disease, immunodeficiency [HIV infection or other]), chronic cardiovascular disease, and chronic liver disease), complications, hospital stay, seasonal influenza, and antiviral treatment. The chi-square test and Fisher’s exact test were used for categorical variables and the median test for continuous variables.

The length of stay was calculated as from the first day of hospital admission in patients with community-acquired influenza and from the onset of influenza symptoms in patients with nosocomial influenza. To assess the relationships between the dependent variable (nosocomial influenza cases Yes/No) and the independent variables studied, a case-case bivariate analysis was made. Possible interactions between independent variables were analyzed by logistic regression. Independent variables were checked for collinearity using the variance inflation factor. Because there were differences between hospitals in nosocomial influenza cases, a mixed-effects logistic regression model with the variable hospital as a random intercept was constructed to estimate the crude and adjusted odds ratios (OR) and their corresponding 95% confidence intervals (CI). A multivariate analysis was performed using the mixed-effects logistic regression model that included the variables described. The final model was selected using the backward procedure, with a cut-off point of *p* < 0.2.

### Ethics approval and consent to participate

As this study was undertaken as part of a national surveillance system, ethical approval was not required [14].

## Results

A total of 1722 patients aged ≥18 years hospitalized with laboratory-confirmed influenza were recorded: 805 (46.7%) were aged 18–64 years and 917 (53.3%) ≥ 65 years and 743 (43.1%) were female. During the six seasons studied, 1480 (85.9%) patients were infected with influenza A viruses (572 H_1_N_1_ and 331 H_3_N_2_) and 241 (14%) with influenza B viruses.

Of the 1722 patients, 96 (5.6%) were admitted to a hospital for a reason other than acute respiratory infection and developed ILI symptoms ≥48 h after admission and were classified as nosocomial influenza. Of these 96 cases, 35 (36.5%) were aged 18–64 years, 22 (22.9%) were aged 64–74 years, 39 (40.6%) ≥ 75 years and 43 (44.8%) were female. Eighty (83.3%) patients presented ≥1 influenza risk factor and 36 (37.5%) had received the influenza vaccine (Table 1).

**Table 1:** Demographic characteristics and comorbidities of patients with community-acquired or nosocomial influenza, Catalonia (Spain)

Variables	Hospital acquired (*N* = 96)	Community acquired (*N* = 1626)	Crude OR (95% CI)	*P* value
Age				
18–64	35 (36.5%)	770 (47.4%)	Ref.	
65–74	22 (22.9%)	305 (18.8%)	1.53 (0.88–2.66)	0.13
> =75	39 (40.6%)	551 (33.9%)	1.42 (0.88–2.29)	0.15
Sex				
Female	43 (44.8%)	700 (43.1%)	1.07 (0.70–1.62)	0.75
Male	53 (55.2%)	926 (56.9%)	Ref.	
COPD				
Yes	26 (27.1%)	414 (25.5%)	1.06 (0.68–1.70)	0.79
No	70 (72.9%)	1212 (74.5%)	Ref.	
Obesity				
Yes	14 (14.6%)	168 (10.3%)	1.29 (0.71–2.35)	0.41
No	82 (85.4%)	1458 (89.7%)	Ref.	
Diabetes				
Yes	34 (35.4%)	396 (24.4%)	1.66 (1.07–2.57)	0.02
No	62 (64.6%)	1230 (75.6%)	Ref.	
Chronic renal disease				
Yes	28 (29.2%)	208 (12.8%)	3.05 (1.89–4.92)	< 0.001
No	68 (70.8%)	1418 (87.2%)	Ref.	
Immunodeficiency				
Yes	27 (28.1%)	307 (18.9%)	1.96 (1.21–3.17)	0.01
No	69 (71.9%)	1319 (81.1%)	Ref.	
Heart disease				
Yes	38 (39.6%)	469 (28.8%)	1.67 (1.09–2.57)	0.02
No	58 (60.4%)	1157 (71.2%)	Ref.	
Liver disease				
Yes	8 (8.3%)	1034 (6.4%)	1.57 (0.73–3.37)	0.25
No	88 (91.7%)	1522 (93.6%)	Ref.	
Virus type				
A	79 (82.3%)	1401 (86.1%)	Ref.	
B	17 (17.7%)	224 (13.8%)	1.49 (0.86–2.59)	0.15
C	0 (0.0%)	1 (0.1%)	–	–
Seasonal influenza vaccination				
Yes	36 (37.5%)	412 (25.6%)	1.62 (1.05–2.52)	0.03
No	60 (62.5%)	1198 (74.4%)	Ref.	

*OR* Odds ratio, *CI* Confidence interval

There were differences between nosocomial influenza patients and those who were not. Nosocomial influenza cases had a higher frequency of age 65–74 years (22.9% vs. 18.8%, *P* < .13), and ≥ 75 years (40.6% vs. 33.9%, *P* < .15), diabetes (35.4% vs. 24.4%, *P* = .02), chronic renal disease (29.2% vs. 12.8% *P* < .001), immunodeficiency (28.1% vs. 18.9% *P* < .001), chronic cardiovascular disease (39.6% vs. 28.8% *P* < .02) and chronic liver disease (8.3% vs. 6.4% *P* = .25). Influenza vaccination uptake was higher in nosocomial influenza patients (37.5% vs. 25.6%, *P* = .03) (Table 1).

Likewise, nosocomial influenza patients more frequently received antiviral treatment within 48 h of symptom onset (67.4% vs. 23.9%, *P* = .01) and had a lower frequency of pneumonia (54.2% vs. 76.4%, *P* < .001). However, nosocomial influenza patients required more days of hospital stay (median: 13 (2–76) vs. 9 (0–137), *P* = .001) and a higher mortality rate was observed (18.8% vs. 12.6%, *P* < .02) (Table 2). No interaction was found between the variables investigated, and there was no collinearity between the variables.

**Table 2:** Antiviral treatment, complications and risk of death in patients with community-acquired and nosocomial influenza, Catalonia (Spain)

Variables	Hospital acquired (*N* = 96)	Community acquired (*N* = 1626)	Crude OR (95% CI)	*P* value
Antiviral treatment				
Yes	89 (92.7%)	1485 (91.3%)	1.06 (0.48–2.34)	0.89
No	7 (7.3%)	141 (8.7%)	Ref.	
Antiviral treatment				
≤ 48 h before symptom onset	62 (67.4%)	375 (23.9%)	2.94 (1.30–6.66)	0.01
> 48 h before symptom onset	23 (25.0%)	1051 (67.1%)	0.42 (0.18–1.00)	0.05
No	7 (7.6%)	141 (9.0%)	Ref.	
Hospital stay; median (range)	13 (2–76)	9 (0–137)		0.001
Hospital stay				
0–14 days	55 (57.3%)	1174 (72.3%)	Ref.	
> 14 days	41 (42.7%)	450 (27.7%)	2.04 (1.33–3.14)	< 0.001
Pneumonia				
Yes	52 (54.2%)	1237 (76.4%)	0.34 (0.22–0.53)	< 0.001
No	44 (45.8%)	383 (23.6%)	Ref.	
ARDS				
Yes	33 (34.4%)	626 (39.3%)	0.86 (0.52–1.42)	0.57
No	63 (65.6%)	965 (60.7%)	Ref.	
Multiorgan failure				
Yes	10 (10.6%)	166 (10.5%)	1.10 (0.56–2.19)	0.78
No	84 (89.4%)	1416 (89.5%)	Ref.	
ICU admission				
Yes	31 (32.3%)	561 (34.5%)	1.00 (0.64–1.56)	0.99
No	65 (67.7%)	1065 (65.5%)	Ref.	
Death				
Yes	18 (18.8%)	205 (12.6%)	1.91 (1.11–3.31)	0.02
No	78 (81.3%)	1421 (87.4%)	Ref.	

*ARDS* Acute respiratory distress syndrome, *ICU* Intensive care unit, *OR* Odds ratio, *CI* Confidence interval

In the multivariable regression model, the variables associated with nosocomial influenza cases were chronic renal disease (aOR 2.44 95% CI 1.44–4.15) and immunodeficiency (aOR 1.79 95% CI 1.04–3.06) (Table 3).

**Table 3:** Multivariate logistic regression model of nosocomial influenza risk factors, Catalonia (Spain)

Variables	Adjusted OR (95% CI)	*P* value
Chronic renal disease		
Yes	2.44 (1.44–4.15)	< 0.001
No	Ref.	
Immunodeficiency		
Yes	1.79 (1.04–3.06)	0.03
No	Ref.	
Liver disease		
Yes	1.72 (0.75–3.96)	0.19
No	Ref.	
Antiviral treatment		
≤ 48 h before symptom onset	3.43 (1.45–8.12)	0.01
> 48 h before symptom onset	0.51 (0.21–1.26)	0.15
No	Ref.	
Hospital stay		
0–14 days	Ref.	
> 14 days	2.57 (1.59–4.16)	< 0.001
Pneumonia		
Yes	0.42 (0.26–0.68)	< 0.001
No	Ref.	
ARDS		
Yes	0.60 (0.34–1.05)	0.07
No	Ref.	
Death		
Yes	2.25 (1.18–4.26)	0.01
No	Ref.	

*ARDS* Acute respiratory distress syndrome, *ICU* Intensive care unit, *OR* Odds ratio, *CI* Confidence interval

Nosocomial influenza cases were a risk factor for death (OR 1.86 95% CI 1.07–3.23). Furthermore, in the multivariable regression model the risk of death was slightly higher (aOR 3.26 95% CI 1.53–6.92) after adjustment for the other variables in the model (Table 4).

**Table 4:** Multivariate logistic analysis of nosocomial influenza as a risk factor for death

	Death (*N* = 223)	No death (*N* = 1499)	Crude OR (95% CI)	*P* value	Adjusted OR (95% CI)	*P* value
Hospital acquired	18 (8.1%)	78 (5.2%)	1.86 (1.07–3.23)	0.03	3.26 (1.53–6.92)	0.002
Community acquired	205 (91.9%)	1421 (94.8%)	Ref.		Ref.	

Adjusted for age, antiviral treatment, pneumonia, multiorgan failure, intensive care unit, renal failure, immunodeficiency and chronic liver disease

## Discussion

This study, based on the surveillance of hospitalized cases of severe laboratory-confirmed influenza, shows that 5.65% of influenza cases in acute hospitals were due to nosocomial transmission and were associated with chronic diseases and a high risk of death. The results highlight the importance of the nosocomial transmission of influenza in acute hospitals in a study with a multicenter design, a large number of patients and an extended study period of six consecutive influenza seasons.

Our results are consistent with other studies that show sporadic nosocomial cases of influenza are a public health problem in acute-care hospital settings. Enstone et al. in the United Kingdom detected 30 nosocomial cases (2.0%) in 1520 hospitalized patients with influenza from 75 hospitals [15]; Macesic et al. in Australia recorded 26 nosocomial cases (4.3%) in 598 hospitalized patients with influenza from 15 hospitals [16]; and Álvarez-Lerma et al in Spain documented 224 nosocomial cases (9.3%) in 2421 hospitalized patients with influenza from 148 intensive care units [17]. Patients with hospital-acquired influenza infection had a profile that differed from that of patients with community-acquired infections; they were older, more likely to have chronic diseases (diabetes, chronic renal disease, immunodeficiency and chronic cardiovascular disease) and had higher mortality (18.8%). Similarly, other studies present poor outcomes. While the mortality of nosocomial cases in the study by Enstone et. al was 26.7% [15], in the study by Álvarez-Lerma et al. it was 39.2% [17]; in both studies mortality was notably higher than that due to community-acquired influenza [15, 17]. Compared with community-acquired influenza cases, we found a significantly longer stay in patients with nosocomial influenza. Nosocomial influenza has previously been associated with longer hospitalization as well as increased use of diagnostic resources and treatments [5].

Even though vaccination rates were very low in patients with both hospital-acquired and community-acquired influenza virus infection, a higher proportion of nosocomial influenza patients had been vaccinated. This has been observed in other studies [15–17] and may be related to the greater number of comorbidities (COPD, diabetes, chronic renal disease, immunodeficiency and chronic cardiovascular disease) for which vaccination is recommended [18].

As with nosocomial influenza outbreaks [19, 20], sporadic nosocomial cases of influenza detected by surveillance during the influenza season may have special interest, as they could suggest failures in hospital infection control. The case series in this study was detected in settings where clinical management and infection control precautions were determined by guidelines [21], and with data recorded by experienced public health staff [4].

There is no standardized definition of nosocomial influenza infection. The mean hospital stay before symptom onset in this study was 14.3 days and we applied a cutoff point of ≥2 days after admission in nosocomial cases. This case definition was based on a review of health care-associated influenza that showed a median threshold delay between admission and symptom onset of 48 h (range: 24–96 h) [22, 23], suggesting that the erroneous inclusion of community cases of influenza as nosocomial cases is quite improbable. Nevertheless, a standardized definition is lacking, and this should be resolved in the future.

Health care-associated influenza cases could signal failures in hospital infection control, as transmission may have been due to an infectious health care worker [24] or to the contaminated hands of a health care worker [5]. However, infection by other patients could also have occurred. Munier-Marion et al. reported that hospitalizations in double-occupancy rooms are an important risk factor for hospital-acquired influenza [25]. Another possibility may be transmission from patients’ visitors [5, 25].

Strategies to reduce influenza transmission in health facilities should include different methods. Patients with risk factors should be vaccinated before the start of the influenza season. In addition, all their contacts and health care workers should also be vaccinated. Barrier precautions, compliance with hand hygiene and exclusion of sick workers should also be ensured. The influenza vaccine is recommended and funded for older people and risk groups in most European countries, although its effectiveness is not absolute [18]. Reducing transmission from advising of not working or not visiting hospitals when people are sick and applying barrier precautions has some limitations due to the possibility of influenza transmission before the onset of symptoms or with minimal symptomatology [24]. Nevertheless, together these measures may help reduce the number of acquired-hospital influenza cases. Furthermore, the study suggested a high risk of death in elderly patients with nosocomial influenza with chronic renal disease or immunosuppressive diseases and those with stem cell transplantation or cancer. Health care workers caring for these patients should be vaccinated every season and mandatory vaccination may be considered.

Our study is based on a case-case analysis of patients hospitalized with severe laboratory-confirmed influenza. The strengths of the study include the large number of patients hospitalized for influenza, the multicenter design, the uniform patient screening by hospitals, the diagnostic confirmation of all patients and the extended study period of six consecutive influenza seasons.

However, the study has also limitations. Some patients may have been discharged before the onset of hospital-acquired influenza. Furthermore, some patients may have minor symptoms which might not be detected and therefore the real number of nosocomial cases might have been underestimated.

## Conclusions

This study shows that nosocomial transmission is a recurrent problem and the total effect of nosocomial influenza may be underestimated. These results suggest the need to improve infection control guidelines and the vaccination of health care workers and ensure clinical suspicion of influenza in high risk areas. In addition, people should be recommended not to visit hospitals when they are ill.

## Data Availability

The datasets used and/or analyzed during this study are available from the corresponding author on reasonable request.

## Abbreviations

ARDS: Acute respiratory distress syndrome
BMI: Body mass index
COPD: Chronic obstructive pulmonary disease
ICU: Intensive care unit
ILI: Influenza-like-illness
RT-PCR: Polymerase chain reaction
